# Diagnosis and Management of Oncologic Emergencies

**DOI:** 10.5811/westjem.2018.12.37335

**Published:** 2019-02-14

**Authors:** Sarah Klemencic, Jack Perkins

**Affiliations:** Virginia Tech Carilion School of Medicine, Department of Emergency Medicine, Roanoke, Virginia

## Abstract

Oncologic emergencies may be seen in any emergency department and will become more frequent as our population ages and more patients receive chemotherapy. Life-saving interventions are available for certain oncologic emergencies if the diagnosis is made in a timely fashion. In this article we will cover neutropenic fever, tumor lysis syndrome, hypercalcemia of malignancy, and hyperviscosity syndrome. After reading this article the reader should be much more confident in the diagnosis, evaluation, and management of these oncologic emergencies.

## INTRODUCTION

Oncologic emergencies are common in emergency medicine (EM). However, they may not often present to emergency departments (ED) that do not serve a robust oncology population. Furthermore, some oncologic emergencies can be subtle in presentation and may be overlooked, contributing to increased morbidity and mortality. We have selected four of the most important oncologic emergencies to review. We will highlight pearls and pitfalls for the emergency physician (EP) so that the recognition, evaluation, and management of these conditions will result in better patient outcomes.

## NEUTROPENIC FEVER

Neutropenic fever (NF) is one of the most well-known oncologic emergencies. Up to 80% of patients receiving chemotherapy for hematologic malignancies will develop NF at least once during the course of therapy.^1^–^3^ Patients with solid tumors are reported to develop NF at a rate of 10–50% during the course of chemotherapy.^1^–^3^ The likelihood of fever increases with the duration and the severity of neutropenia as well as the rate of decline of the absolute neutrophil count (ANC).^4^ The ANC nadir is often 7–10 days after the conclusion of chemotherapy.^5^ NF is defined as a single oral or axillary temperature of ≥ 38.3°Celsius (C) (101°Fahrenheit [F]) or a temperature ≥ 38.0°C (100.4°F) sustained over 60 minutes in a patient with an ANC < 500/μL (microliter).^5^ Neutropenia can be characterized as mild, moderate, severe, or profound (Table 1).^5^, ^6^

While EPs should be most concerned with bacterial etiologies of NF, it is actually uncommon for a definite etiology to be determined for an episode of NF.^7^,^8^ Only 20–35% of episodes of NF are due to a clinically documented infection (i.e., source identified by culture, antigens, or other testing modalities).^2^–^4^, ^7^–^8^ This should be expected since NF may be due to the underlying malignancy itself (e.g., leukemia), mucositis, toxicity of the chemotherapeutic agents, or a host of other etiologies.^2^, ^3^ If a bacterial source is the culprit, it is most likely to be endogenous flora from the gut (e.g., *Escherichia coli*,* Enterobacter*), skin (e.g., *Staphylococcus*, *Streptococcus*), or respiratory tract (e.g., *Streptococcus*).^2^–^4^, ^7^–^8^ The past few decades have seen a change in the bacterial epidemiology associated with NF. Gram-positive bacterial infections (e.g., *Staphylococcus*, *Streptococcus)* have become at least as likely as gram-negative infections (e.g., *Escherichia coli, Pseudomonas)* due to a rising incidence of indwelling catheters and a higher community burden of *Staphylococcus.*^2^–^4^, ^7^–^9^ Additionally, the incidence of *Clostridium difficile (C. diff)* and resistant gram-negative pathogens is increasing.^10^ A fungal etiology is unlikely if it is the patient’s first episode of NF; however, this risk increases if the patient is taking empiric antibiotics, receiving total parenteral nutrition, or has concurrent mucositis.^11^

Once a patient is identified as having NF, it is incumbent upon the EP to proceed systematically in terms of diagnostic evaluation, antibiotic administration, and disposition. Standard initial testing should include a complete blood count (CBC) with manual differential, complete metabolic panel (CMP), two sets of blood cultures (including one from an indwelling line if applicable), urinalysis and culture, and chest radiograph (CXR) (two views preferred).^5^ If the patient has diarrhea, consider adding stool cultures and *C. diff* testing. Keep in mind that in the winter influenza testing should be regarded as standard for NF evaluation. It is important to keep in mind that the neutropenic patient will not be able to mount a robust inflammatory response, and thus the sensitivity of a CXR will decrease.^12^–^14^

Broad-spectrum antibiotics should be administered within 60 minutes once NF is identified and appropriate cultures have been obtained.^5^,^15^–^16^ The choice of empiric antibiotic (e.g., cefepime, meropenem) will vary based on the institution according to the local antibiogram. Refer to Table 2 for common empiric regimens for NF.^17^–^22^

Empiric coverage for gram-positive organisms (e.g., vancomycin) is indicated in patients who are hypotensive, have a skin and soft tissue source, are currently taking a fluoroquinolone or trimethoprim/sulfamethoxazole, or who have an indwelling line.^5^ While NF is most certainly a medical emergency requiring timely source assessment and delivery of broad-spectrum antibiotics, it is no longer standard to admit all NF patients to the hospital.^23^–^26^ In fact, recent literature suggests that EPs are not familiar with the most recent NF guidelines both in terms of antibiotic deployment (i.e., when vancomycin is recommended) and disposition.^23^–^24^ Much as in other diseases seen in EM, a continuum exists in NF such that some patients will be at much higher risk of developing sepsis and its related morbidity and mortality.

Any decision on disposition of the NF patient should be made in conjunction with the patient’s oncologist or the on-call oncologist. Even if the patient is clearly in need of admission, early oncology input is essential as they may well have pertinent clinical information that is not available in the electronic medical record regarding prior episodes of NF for that patient, current chemotherapy regimen and side effects, and potential for more unusual pathogens (e.g., fungal, viral, parasitic).^26^ While all NF patients were commonly admitted in the past, disposition of patients with NF is no longer straightforward as not all patients may require admission to the inpatient setting.^26^ In addition to the cost and resource utilization (i.e., occupied inpatient bed) associated with an inpatient stay, there is risk to the patient with neutropenia in being admitted to the hospital with subsequent exposure to nosocomial pathogens.^27^ Any NF patient with sepsis requires admission to the hospital as do those patients with significant co-morbid illness (e.g., congestive heart failure, chronic obstructive pulmonary disease) or an unstable social situation precluding reliable follow up.^24^–^26^ Select patients (i.e., not septic, no major co-morbid illness, stable social situation) may be suitable for outpatient management of NF. Most experts recommend using the Multinational Association for Supportive Care in Cancer score (MASCC) for assisting with disposition decisions (Table 3).^28^

It is important to note that a higher MASCC score is associated with better outcomes. Scores ≥ 21 are considered “low risk” and these patients may be suitable for outpatient management.^28^ In the past few years, a new scoring system referred to as CISNE (Clinical Index of Stable Febrile Neutropenia – see Table 4)^29^ has been developed, and early literature comparing MASCC and CISNE has been promising in terms of equivalence.^30^–^31^ However, it is important to note that the clinical practice guidelines were developed before CISNE was validated, and use of this tool should be considered with consultation from the patient’s oncologist.

## TUMOR LYSIS SYNDROME

Tumor lysis syndrome (TLS) is a rare but potentially deadly metabolic crisis with an estimated mortality of 29–79%.^32^–^35^ With early and aggressive intervention, the mortality rate in TLS can be impacted significantly. TLS is the most frequently encountered metabolic complication of hematologic malignancy by an EP.^32^–^37^ Tumor lysis syndrome occurs due to the liberation of intracellular components into the circulation.^32^ It rises in incidence with malignancies that have rapid cell turnover (e.g., hematologic malignancies).^33^ While TLS most commonly occurs subsequent to chemotherapy, it may occur spontaneously in patients with hematologic malignancies (especially acute leukemias).^34^,^35^ Patients with solid tumors rarely develop TLS after chemotherapy.^34^ Those with baseline renal dysfunction, elderly patients with comorbidities, and patients taking multiple medications are at greater risk of developing TLS.^32^–^35^ Presenting symptoms can include fatigue, signs of dehydration, seizures, cardiac dysrhythmia, nausea and vomiting.^36^–^37^

The predominant intracellular contents released systemically include potassium, phosphate, and uric acid. Consequently, laboratory values may reflect hyperkalemia, hyperphosphatemia, hypocalcemia, and hyperuricemia.^36^ Initial testing should include CBC with differential, CMP, lactate dehydrogenase, uric acid, phosphate, total and ionized calcium levels, and urinalysis. An electrocardiogram (ECG) should also be obtained given the potential for electrolyte derangements.^32^–^39^ Hyperkalemia poses the most immediate threat to the patient and is secondary to massive cellular breakdown, which overwhelms the kidneys. Hyperkalemia may be worsened by patient use of potassium-sparing medication, metabolic acidosis or prior renal insufficiency or failure. Phosphorous is present in malignant cells fourfold compared to normal cells; therefore, lysis of malignant cells releases large quantities of phosphate into the circulation, which ultimately binds with calcium to form calcium phosphate crystals.^39^ The crystals deposit into soft tissue and can contribute to complications such as urinary obstruction, iritis, and skin lesions.^36^–^38^ Hypocalcemia secondary to phosphate binding may cause symptoms of anorexia, vomiting, seizures or cardiac arrest.^41^ The Cairo-Bishop criteria are preferred to diagnose TLS (Table 5). The diagnosis of TLS can be made before the development of acute kidney injury (AKI) and this is the best time for intervention.^36^–^40^ Patients with TLS who develop AKI have a higher rate of mortality.^41^

Once TLS is identified, initial interventions consist of aggressive intravenous fluid (IVF) administration and correction of electrolyte abnormalities.^39^–^43^ Isotonic fluid resuscitation is recommended with a goal of at least 2000–3000 L/m^2^/day (liters per meters squared per day) for adults and children. (Use goal of 200 milliters per kilogram [kg] per day for children less than 10 kg)^39^–^43^ Hyperkalemia secondary to TLS should be a treatment priority and should proceed similarly as with other hyperkalemic patients.^39^–^43^ Ultimately, dialysis may be required for severe or refractory cases of TLS to treat renal failure as well as severely elevated uric acid, potassium or phosphate levels.^39^–^42^ Phosphate binders such as aluminum hydroxide (300–600 mg [milligram] oral dose) may be used to treat excess phosphorus in stable patients who have a phosphate level ≥ 6.0 mg per deciliter (dl). 40 Symptomatic hypocalcemia (e.g., seizures, tetany or cardiac dysrhythmias) should be treated with calcium gluconate one gram intravenously.^40^–^41^ This dose may be repeated as required for symptom management. It is important to emphasize that asymptomatic hypocalcemia should not be treated as the additional calcium may cause calcium phosphate precipitation and acute obstructive uropathy.^39^–^41^

Additionally, hyperuricemia should be addressed to prevent uric acid nephropathy as it may lead to decreased filtration rate and crystal obstruction.^44^ Allopurinol is effective in the prevention of uric acid production; however, it does not decrease uric acid already present, and so is less effective in treating TLS.^44^–^45^ Rasburicase, a recombinant urate oxidase, has shown good promise when used for hyperuricemia.^44^–^47^ Humans lack urate oxidase, which metabolizes uric acid to the more soluble allantoin, which can then be renally excreted.^44^–^45^ Studies have shown rasburicase is more effective in lowering serum uric acid levels in patients with TLS compared to allopurinol, is well tolerated by patients, and does not require adjustment for changes in creatinine.^45^–^46^ The recommended dose is 0.2 mg/kg by IV therapy. Of note, rasburicase is contraindicated in patients with history of glucose-6-phosphate dehydrogenase deficiency.^47^ Management of TLS requires coordination with the patient’s oncologist and frequent laboratory testing and intensive nursing care, which is often why these patients necessitate an intensive care unit (ICU) admission.^32^

## HYPERCALCEMIA OF MALIGNANCY

Hypercalcemia is seen in 10–30% of patients with malignancy and is most commonly associated with breast cancer, lung cancer, non-Hodgkin’s lymphoma and multiple myeloma, although it may be seen with any malignancy.^48^–^55^ Twenty percent of malignancy-related hypercalcemia is secondary to bony metastases, and it should be noted that the incidence of hypercalcemia increases with advanced disease and portends a poor prognosis.^50^ Multiple pathways lead to hypercalcemia of malignancy; however 80% can be attributed to parathyroid-related protein (PTHrP) activity.^51^ PTHrP increases bone resorption via osteoclast activity and enhances calcium resorption in the renal tubule. 51 Importantly, an EP should consider malignancy in any patient (without a known diagnosis of malignancy) presenting with hypercalcemia of unclear etiology.^48^–^50^ In these patients, the likelihood of an underlying malignancy rises in direct correlation to the degree of hypercalcemia.^48^ Importantly, symptoms are related to the rate of rise of serum calcium and are not solely based on the absolute value.^51^

The symptoms of hypercalcemia are vague and often reflect symptoms associated with significant volume depletion due to the osmotic diuresis associated with hypercalcemia.^51^ The most common symptoms are anorexia, nausea, vomiting and constipation, but may include malaise, polyuria, polydipsia, lethargy, confusion, and even coma.^48^–^52^ Laboratory analysis should include both a total calcium and ionized calcium level when possible. If ionized calcium values are unavailable, a corrected calcium value can be calculated as follows: Corrected calcium level = measured calcium level + (0.8 × [4.0 - serum albumin level {g/dl}]) The EP should also send a full CMP, CBC, a magnesium level, and phosphate level. Parathyroid and PTHrP testing are useful for the oncologist, but are not indicated in the emergent setting.^52^–^55^ An ECG may show prolonged PR, widened QRS, shortened QT, and ventricular dysrhythmias.^52^–^55^ Immediate treatment for calcium levels below 12 mg/dl can be deferred. Patients with moderate hypercalcemia with levels of 12–14 mg/dl should be treated based on clinical judgment and symptom control as these levels may have been reached either acutely or subacutely and may even be well tolerated. Nonetheless, any patient with a serum value >14mg/dl is generally symptomatic and should receive an intervention to lower the level.^52^,^55^ Cardiac arrest may occur with levels >15 mg/dl. 49

Initial emergent management of hypercalcemia involves aggressive IVF administration with an initial bolus of 1000–2000 ml of isotonic fluid followed by an infusion rate of 200–300 ml/hr (milliliters per hour) to achieve urine output of 100–150 ml/hr.^49^, ^52^–^55^ Loop diuretics will decrease serum calcium levels, and studies have shown high doses are required to be effective; therefore, use should only be considered in the euvolemic patient or those with concurrent volume overload.^49^, ^52^–^55^ Bisphosphonates lower calcium levels by inhibiting osteoclasts and stabilize the bone matrix by binding to calcium phosphate. These medications are renally excreted, and the dose will need to be adjusted based on renal function.^49^ Complications may include self-limited infusion-related fever or AKI.^49^ Calcitonin decreases bone resorption and enhances urinary excretion of calcium and may be employed via intramuscular or IV route.^52^–^54^ The effects are rapid though transient with poor efficacy; therefore, utilization should be considered in adjunct with bisphosphonates when rapid reduction of serum calcium is required.^49^

Glucocorticoids are most effective in patients with Hodgkin’s or non-Hodgkin’s lymphoma or any malignancy that overproduces calcitriol.^52^–^53^ Glucocorticoids inhibit conversion of 25-hydroxyvitamin D to calcitriol, decreasing gut absorption and renal reabsorption of calcium. These medications have slow onset of action and dosing is uncertain, though a recommended dose is IV hydrocortisone 200–300 mg/day.^52^–^54^ Hemodialysis is reserved for those patients with oliguric renal failure.^52^–^54^ Most patients who have mild symptoms, or are asymptomatic with a serum calcium < 14 mg/dl, are good candidates for outpatient management after discussion with their oncologist.^49^, ^54^ Patients in the moderate or severe range of hypercalcemia should be considered for monitored or ICU admission depending on presentation, labs and clinical judgment. Finally, given the significant mortality associated with this presentation, it is important to establish goals of care with the patient and his or her oncologist.

## HYPERVISCOSITY SYNDROME

Hyperviscosity syndrome (HVS) is a rare but potentially catastrophic consequence of increased serum viscosity due to excess serum proteins.^56^–^59^ Hyperviscosity syndrome is the consequence of a significant excess in serum proteins (e.g., Waldenström’s macroglobulinemia [WM] or multiple myeloma) or cellular components (e.g., white blood cells in acute leukemias).^60^ Patients with WM are at highest risk for HVS with 40–90% of HVS cases occurring from WM.^61^ HVS can also be seen in diseases such as multiple myeloma (second most common cause of HVS), leukemia, and polycythemia.^59^ Hyperviscosity syndrome results in relative hypoperfusion, and resultant clinical manifestations represent end-organ dysfunction that may mimic other disease pathology. For example, patients with HVS may complain of visual changes (mistaken for cerebral vascular accident), dyspnea (mistaken for pulmonary embolus or congestive heart failure), or altered mental status (mistaken for sepsis). The classic triad of HVS is mucosal or skin bleeding, visual changes, and focal neurologic deficits; although it is not clear what percentage of patients present with this classic triad.^56^–^60^ The EP should consider this diagnosis in any patient found to have a markedly elevated white blood cell count (i.e., >100 × 10^4^) or hemoglobin (i.e., approaching 20 g/dl) associated with symptoms of hypoperfusion.^61^–^63^ Lab values that will offer the most information include the CMP (total protein and albumin level), CBC (hemoglobin, white blood cell count) with peripheral smear, and coagulation testing (coagulopathy is common in HVS).^63^–^66^ Blood transfusion can significantly worsen HVS and should be avoided if possible. The mainstays of therapy are decreasing serum viscosity through IV fluid resuscitation, plasmapheresis, or leukopheresis.^63^–^67^ Phlebotomy (e.g., polycythemia) or even urgent chemotherapy (e.g., acute leukemia) may also be indicated.^63^–^67^ Evidence for these interventions suggests they do not alter the course of the disease but rather help with symptom alleviation. All patients with HVS will require admission to the hospital and warrant strong consideration for ICU admission.

## CONCLUSION

Oncologic emergencies are becoming increasingly common presentations in the ED both in the community and academic settings. Emergency providers must appreciate the complexity of NF and understand that early, broad-spectrum antibiotics are key to reducing mortality even if the patient does not ultimately get admitted. Tumor lysis syndrome is a subtle but lethal metabolic derangement seen most often in hematologic malignancies that requires aggressive fluid resuscitation and electrolyte management. Hypercalcemia of malignancy heralds a poor prognosis and goals of care should be addressed while providing IV volume resuscitation to counter the osmotic diuresis caused by the hypercalcemia. Finally, hyperviscosity syndrome is especially dangerous as it mimics more common presentations but should be in the differential for any patient with WM, multiple myeloma, severe leukocytosis (i.e., >100 × 10^4^), or a hemoglobin > 20 g/dl. All of these oncologic emergencies require early involvement of oncology for management.

## Figures and Tables

**Table 1:** Degree of neutropenia.

Mild neutropenia	ANC 1000–1500
Moderate neutropenia	ANC 500–999
Severe neutropenia	ANC 100–499
Profound neutropenia	ANC < 100

*ANC*, absolute neutrophil count.

**Table 2:** Common empiric antibiotic selections for neutropenic fever.

Cefepime	Meropenem	Piperacillin-tazobactam	Ceftazidime
2 grams IV Q8	1 gram IV Q8	4.5 grams IV Q6–8	2 grams IV Q8

*IV*, intravenous; *Q8*, every 8 hours; *Q6–8*, every 6–8 hours.

**Table 3:** Multinational Association for Supportive Care in Cancer (MASCC) scoring tool.

Characteristic	Weight (points)
Burden of febrile neutropenia with no or mild symptoms	5
No hypotension (systolic blood pressure > 90 mmHg)	5
No chronic obstructive pulmonary disease	4
Solid tumor or hematological malignancy with no previous fungal infection	4
No dehydration requiring parenteral fluids	3
Burden of febrile neutropenia with moderate symptoms	3
Outpatient status	3
Age > 60 years	2

*mmHg*, millimeters of mercury.

**Table 4:** Clinical index of stable febrile neutropenia (CISNE).

Characteristic	Score (points in parentheses)
Eastern cooperative oncology group performance status	< 2(0) or ≥ 2 (+2)
Stress-induced hyperglycemia (blood glucose ≥ 121 mg/dl)	No (0) or Yes (+2)
COPD	No (0) or Yes (+1)
Cardiovascular disease history (valvular disease, cardiomyopathy, cor pulmonale)	No (0) or Yes (+1)
NCI mucositis grade ≥ 2	No (0) or Yes (+1)
Monocytes	≥ 200 μL (0) or < 200 μL (+1)

*mg/dl*, milligrams per deciliter; *COPD*, chronic obstructive pulmonary disease; *NCI*, National Cancer Institute; *μL*, microliters.

Used in adult outpatients with solid tumor, fever, and ANC δ 500.

**Table 5:** Cairo-Bishop criteria for clinical and laboratory tumor lysis syndrome.

Two or more of the following criteria either three days prior to or seven days after chemotherapy: Uric acid: ≥ 8mg/dL or 25% increase from baseline Potassium: ≥ 6 mEq/L or 25% increase from baseline Phosphorous: ≥ 6.5 mg/dL for children or ≥ 4.5mg/dL for adults or 25% of increase from baseline Calcium: ≤ 7mg/dL or 25% decrease from baseline
Clinical tumor lysis syndrome Laboratory tumor lysis syndrome plus one or more of the following: Creatinine > 1.5 times the upper limit of age-adjusted reference range Cardiac dysrhythmia or sudden death Seizure

mg/dl, milligrams per deciliter; mEq/L, milliequivalents per liter.
